# A review on the anesthetic management of obese patients undergoing surgery

**DOI:** 10.1186/s12871-022-01579-8

**Published:** 2022-04-05

**Authors:** Rimanatou Seyni-Boureima, Zongze Zhang, Malyn M.L.K Antoine, Chrystal D. Antoine-Frank

**Affiliations:** 1 Department of Anaesthesiology, Zhongnan Hospital, Wuhan University, East Lake Road, 430071 Wuhan, Hubei China; 2 Department of Endocrinology, Zhongnan Hospital, Wuhan University, East Lake Road, 430071 Wuhan, Hubei China; 3 Department of Anatomical Sciences, St. George’s University, True Blue,Grand Anse, West Indies St. George, Grenada

**Keywords:** Obesity, Anesthesia, Surgery, Body Mass Index

## Abstract

There has been an observed increase in theprevalence of obesity over the past few decades. The prevalence of anesthesiology related complications is also observed more frequently in obese patients as compared to patients that are not obese. Due to the increased complications that accompany obesity, obese patients are now more often requiring surgical interventions. Therefore, it is important that anesthesiologists be aware of this development and is equipped to manage these patients effectively and appropriately. As a result, this review highlights the effective management of obese patients undergoing surgery focusing on the preoperative, perioperative and postoperative care of these patients.

## Background

According to the World Health Organization (WHO), the prevalence of obesity has significantly increased since 1975. Based on this source, in 2016, approximately 13% of the world’s population were labeled as obese [1]. In addition, over the past few decades, the prevalence of obesity has been steadily increasing in the United States [2, 3]. The Centre for Disease Control and Prevention (CDC) declares that about 35.7% of adults in the United States are now obese [4]. Obesity is associated with comorbidities such as hypertension, type 2 diabetes mellitus and coronary artery diseases. Furthermore, patients who are overweight or obese may also experience dyslipidemia, obstructive sleep apnea (OSA) liver and gallbladder diseases, osteoarthritis, cancers and reproductive and psychological disorders. It is also important to note that obesity is a major risk factor for asthma development and higher prevalence of this disease is commonly seen in obese and overweight persons as compared to non-obese individuals [5]. Due to this myriad of concomitant diseases and complications that accompany obesity, the management of obese patients, especially those undertaking surgical procedures, is now becoming increasingly challenging. The presence of these conditions at some point may require surgical intervention and therefore, anesthesiologists are frequently faced with the challenge of effectively managing obese patients along with their pre-existing comorbidities [6].

According to the literature, obesity with its related comorbidities, significantly increase the risk for preoperative, intraoperative and postoperative surgical complications [7]. Preoperatively, most of the complications observed are associated with the respiratory system as obese patients are more prone to experiencing decreased lung volume, lung collapse, abnormalities in lung and chest wall compliance in addition to varying degrees of hypoxemia [8]. Intraoperative complications are associated with increased block failures [8], peripheral nerve injuries, thrombotic complications and difficulties with airway management and fluid administration [9]^,^. Postoperatively, obese patients also exhibit an increased risk for developing myocardial infarctions, wound and urinary tract infections, deep venous thrombosis (DVT) and nerve injuries [7]. There may also be challenges encountered in finding the appropriate drug doses for induction and maintenance in these patients [10].

As a result, it is imperative that the anesthetic team acquire adequate and relevant knowledge for the effective management of obese patients undertaking different types of surgery. It is also extremely important that the patients be appropriately accessed preoperatively for the identification of anesthesia related risk factors so that the team can adequately prepare for the proper management of any complication that may arise throughout the course of surgery. This paper will therefore discuss the clinical management of obese patients undergoing surgery as a means of providing anesthesiologists with the necessary information needed to properly prepare and manage these patients before, during and after surgery.

### Main Text

#### Definition of Obesity

Obesity is defined by body mass index (BMI). BMI is calculated by dividing body weight measured in kilograms by height measured in meters squared. A BMI ranging between 25.0 and 29.9 kg/m^2^ is used to define overweight while obesity is defined by a BMI of 30 kg/m^2^ or greater (see Table 1). For individuals between the ages of 2 to 18 years the percentile scale is utilized for defining obesity rather than BMI [11, 12]. Fat in the body can be described differently. Increased fat deposition in the lower regions of the body is described as peripheral obesity whereas higher abdominal or visceral fat deposition is considered central obesity [13]. A waist circumference of more than 88 cm in women and 102 cm in men or a waist to height ratio of more than 0.55 defines central obesity [14, 15]. Central obesity is most commonly associated with pathological conditions [16, 17]. Fat tissues that are distributed in the central region of the body are more likely to produce inflammatory mediators which may place obese patients at greater risks for obesity associated metabolic diseases [18]. Patients exhibiting central obesity also show increased risks for experiencing perioperative complications [19].

**Table 1:** World Health Organization classification of obesity [20]

Body mass index (kg.m^2^)	Classification
< 18.5	Underweight
18.5 – 24.9	Normal
25.0 – 29.9	Overweight
30.0 – 34.9	Obese Class 1
35.0 – 39.9	Obesity Class 2
> 40.0	Obese Class 3 (previously ‘morbid obesity’)

### Pathophysiology of Obesity

Obesity is described as a multifactorial disease caused by the interplay of various environmental, genetic and hormonal factors. Excessive intake and decreased expenditure of calories can contribute to the development of obesity. Energy balances within the body is partly controlled by interaction between the hypothalamus and peripheral tissues and organs [21]. Genes such as the beta-3-adrenergic receptor gene, peroxisome-proliferator-activated receptor gamma 2 gene, chromosome 10p, and melanocortin-4 receptor gene have all been identified as genetic contributors to the pathogenesis of obesity. Adipocytes produce hormones called adipokines primarily tumour necrosis factor-alpha (TNF-α), interleukin-6 (IL-6), leptin and adiponectin. TNF-α promotes insulin resistance and inflammation of blood vessels. IL -6 also promotes inflammation, impairs host immunity and induces tissue injury [22]. Leptin decreases appetite and its deficiency is rarely observed in humans; however, obese individuals are often described as being leptin-resistant. Adiponectin promotes insulin sensitivity, reduces inflammation, and inhibits atherogenic activities. It is observed that adipose tissues of obese individuals exhibit a decreased expression of adiponectin messenger RNA [23]. The presence of central obesity promotes inflammation which subsequently leads to insulin resistance and endothelial dysfunction as increased levels IL-6, TNF-α and C-reactive protein and decreased levels of adiponectin and interleukin-10 are observed [22].

Obesity seems to be associated with lower levels of vitamins such as vitamins A,D and E. Furthermore, deficiencies in the B vitamins have also been associated with obesity [24]. Minerals such as zinc, iron, calcium and selenium, when deficient, can also contribute to weight gain and subsequent obesity. Evidence suggests that persons who are morbidly obese may exhibit lower levels of vitamins C and E [25] and further evidence also demonstrates generally lower levels of beta-carotene and vitamin C in adults that are overweight or obese [26]. These vitamins and minerals may work to prevent obesity in different ways which may be achieved by inhibiting adipogenesis, inducing apoptosis of adipocytes, regulating the production of certain hormones like leptin, decreasing oxidative stress and inflammation, inhibiting lipogenesis and promoting lipolysis [27]. Therefore, the deficiency of micronutrients should also be given special consideration when investigating potential causes of obesity.

### Anatomical airway changes in obese patients

Normal respiration may be affected in obese patients due to the excessive amount of adipose tissue that is deposited in areas like the chest walls, ribs, diaphragm and abdomen [28]. For normal respiration to occur, the diaphragm contracts, displacing abdominal contents inferiorly and anteriorly. The external intercostal muscles also contract pulling the ribs superiorly and anteriorly [29]. In individuals that are obese, these normal actions are mechanically impeded by the presence of excessive adipose tissue in the thoracic and abdominal regions; their lung compliance is decreased. Measurements of maximal inspiratory pressure (MIP) and maximal expiratory pressure (MEP) can be used to evaluate the strength of respiratory muscles and these measurements are observed to be reduced in individuals that are obese [30]. In addition, when an obese individual lies flat on the back, weight from the abdomen moves superiorly into the thoracic cavity. This compresses and occludes small airways at the lung bases causing laboured ventilation and impairment in the normal function of major respiratory muscles [31, 32].

Various changes in lung volumes are observed in obese patients. The expiratory reserve volume (ERV), functional residual capacity (FRC), and the overall total lung capacity (TLC) are all reduced in individuals that present with obesity. These changes occur due to imbalances in pressures within the lungs, resulting in abnormal lung inflation and deflation [33]. Even though most obese individuals will have a normal arterial partial pressure of oxygen (PaO_2_), it is observed that individuals that are morbidly obese present with mildly widened alveolar-arterial oxygen gradients [P(A-a) O_2_]. This occurs due to the ventilation perfusion imbalances that occur in the lungs of morbidly obese individuals secondary to partial lung collapse. Observations show that the lungs of individuals that are morbidly obese exhibit increased ventilation and perfusion in the upper regions and decreased ventilation and perfusion in the lower regions [28, 34].

### Perioperative Care of Obese Patients undergoing surgery

Obese patients, especially those presenting with comorbidities, may potentially exhibit increased risks for experiencing complications during surgical procedures [14]. The Obesity Surgery Mortality Risk Stratification score (OS-MRS) has been established for the assessment of patients who are undertaking gastric bypass surgery [35]. This score is essential as it helps in the isolation and identification of risk factors that may increase mortality outcomes in obese patients undergoing bariatric surgery. Despite its implications for use in gastric bypass surgeries, this assessment tool may also prove beneficial in assessing obese patients undergoing normal surgeries. Patients with an OS-MRS score of 4-5 should be closely monitored during surgical procedures [14]. As obese patients are prepared for undertaking surgery, it is important that their BMI be calculated and the resulting information be relayed to the operating team so that necessary preparations would be made to accommodate the patient safely and comfortably during surgery. Patients should also be carefully assessed as to identify any pre-existing comorbidities and to determine potential complications that may arise from the surgery [36]. Proper guidance should also be provided through the use of counseling, highlighting necessary modifications such as smoking cessation before surgery and early mobilization after surgery [14] as this helps to limit the occurrence of complications. Before the surgery, proper assessment of major body systems is also important.

#### Respiratory Assessment

Determination of arterial saturation for obese patients undergoing surgery is essential as patients presenting with an arterial PCO_2_ (Partial Pressure of Carbon Dioxide) that is greater than 6 kPa has an increased risk for experiencing complications as some degree of respiratory failure is usually present [37]. While completing the general respiratory assessment, it is also important to query about sleep-disordered breathing which can be done using the STOP-BANG questionnaire. A score of 5 or more obtained from this screening method implies the presence of sleep-disordered breathing [38, 39]. This, therefore, warrants a referral to a specialist prior to surgery. For patients presenting with a score of less than 5, a referral to a specialist may also be necessary if the patient has a history of dyspnea upon exertion, experiences headaches especially in the morning, or presents with ECG changes indicative of right atrial hypertrophy [36]. Patients that present with OSA and an inability to tolerate continuous positive airway pressure (CPAP) also demonstrate increased risk for perioperative respiratory and cardiovascular complications [40].

It is important to note that chances of difficult or failed intubation are much greater in patients that are obese. The measurement of the patients’ neck circumference can be helpful as a neck circumference over 60 cm increases the chances for experiencing difficult intubation [41]. In addition to difficult or failed intubations, difficult bag-mask ventilations are also observed in patients that are obese [36]. As part of the preoperative airway assessment the anaesthesiologists should enquire about the following from the patients’ past medical history: (1) a history of OSA, (2) a history of gastro-oesophageal reflux disease and (3) a history of difficult anaesthesia or airway management. As the preoperative airway assessment is done, it is important to note that patients presenting with a short distance between the chin and the tip of the thyroid cartilage, flattened anterior-posterior craniofacial features, narrowed oropharynx and relative macroglossia are at an increased risk for experiencing airway obstruction when undergoing general anaesthesia. In general, when carrying out a preoperative respiratory assessment in obese patients, the following should be noted (1) the circumference of the patients neck, (2) the distance between the mentum and upper boundary of the thyroid cartilage, (3) the extent of mouth opening and jaw protrusion, (4) neck mobility, (5) the presence of excessive adipose tissue in the cervical region of the neck and (6) and the general features of the patients’ head and face. Assessments should also be carried out for the presence of OSA [42].

#### Cardiovascular Assessment

During the cardiovascular assessment phase, it is important to pay close attention to any features of metabolic syndrome that may be present as this may be a major indication for cardiovascular complications [43]. The use of ECGs are also critical as part of the cardiovascular assessment as they allow for the identification of undiagnosed pre-existing cardiac abnormalities [44]. This is particularly important as obese and overweight patients exhibit an increased risk for developing arrhythmia, especially atrial fibrillation, and ventricular tachycardia, which can be detected by the ECG. Cardiac arrhythmias in obese or overweight patients are usually precipitated by a myriad of factors such as hypoxia and preexisting heart diseases. Mechanical factors such as obstructive sleep apnea may also influence the development of arrhythmias in these patients [45]. Recent evidence suggests an association between obesity and the development of atrial fibrillations [46]. Furthermore, overweight, and obese patients may show a 50% increased risk for developing this arrhythmia [47]. Different contributors such as remodeling of the atrium, increased blood volume, elevated left atrial pressure and neurohormonal factors, amongst others, may significantly influence this occurrence [46]. Hemodynamic changes observed in the obese causes structural and physiological changes within the heart that potentially induce atrial fibrillation. Excess deposition of adipose tissues increases total blood volume which subsequently increases cardiac output (increases mainly due to an increase in stroke volume) [48]. As cardiac output steadily increases, hypertrophy (eccentric or concentric) of the left ventricle eventually occurs [49], subsequently increasing left ventricular filling pressures, therefore, causing diastolic dysfunction. Systolic dysfunction may also ensue following enlargement of the left ventricle [50]. In addition, left atrial hypertrophy occurs causing the pressures and volumes within the left atrium to increase [51]. This therefore causes pulmonary hypertension to develop. Besides, obesity is also associated with OSA which effects can consequently alter autonomic tone due to hypoxia, acidosis, and disturbances in the sleep cycle. Autonomic tone alterations potentially increase pulmonary arterial pressures which subsequently cause right ventricle hypertrophy and eventual ventricular failure [52]. These changes observed in the left and right heart along with the observed hemodynamic changes, significantly contribute to the development and maintenance of atrial fibrillations observed in the obese. Therefore, it is quite important that obese patients be assessed for the presence of atrial fibrillations and other common arrhythmias such as ventricular and supraventricular tachycardia and premature atrial and ventricular contractions. In addition, these patients should be closely monitored for the postsurgical development of arrhythmias especially if the patient has pre-existing heart diseases.

In addition, as part of the cardiovascular assessment phase, the cardiopulmonary exercise testing can be applied as it helps in predicting postoperative prognoses including complications that may arise and the average length of hospital stay that might be required [53, 54]. It is sometimes difficult to measure the blood pressure of obese patients with the use of standardized equipment; therefore, direct arterial monitoring can be employed for the accurate determination of blood pressure measurements [55]. Knowledge on the following conditions may assist in assessing the potential risks of cardiovascular related morbidities: (1) the type of surgery, whether it’s considered high-risk or not, (2) the presence of coronary artery disease, (3) an existing history of congestive heart failure, (4) the presence of cerebrovascular disease, (5) a history of insulin use preoperatively and (6) plasma creatinine levels measuring >2 mg/dl prior to surgery [56].

#### Pre-oxygenation

As compared to non-obese patients, morbidly obese patients may desaturate more quickly during apnoea. As a result, steps should be taken to prevent or reduce the chance for a fall in oxygen saturation after pre-oxygenation. The necessary steps are as follows: (a) when the patients is being pre-oxygenated, an upright head position of about 25 degrees should be maintained [57], (b) while inserting the laryngoscope, oxygen should be passively administered, with the use of a 10 Fr catheter through the nasopharynx, at a rate of about 5 L/min ^−1^ [58] and 3) during pre-oxygenation, the application of 10cmH_2_O of positive end-expiratory pressure (PEEP) should be considered [59]. To reduce the occurrence of pre-oxygenation induced atelectasis,  inspiratory pressure should be maintained at about 55cmH_2_O for 10 s directly following the application of 10cmH_2_O of PEEP [60, 61]. In morbidly obese patients, once the airway is secured the inspired oxygen fraction should be reduced and maintained at about 0.4 [62, 63].

#### Pre-anaesthetic medication

Pre-anaesthetic medications may be considered in obese patients undergoing surgeries as to alleviate surgical complications which can take the form of infections, gastrointestinal disturbances, postsurgical pain, hyper-coagulation and anxiety. Antimicrobials such as cefazolin can be appropriately administered as prophylaxis for the prevention of postsurgical infections [64–66]. Obese individuals with body weight ≥ 120 kg will require a prophylaxis dose of 3 g of cefazolin to curb the risk of surgical-site infections [66]. Nausea and vomiting may be commonly observed gastrointestinal disturbances. As means of preventing postsurgical nausea and vomiting, the preoperative use of dexamethasone combined with ondansetron and haloperidol can be considered [36, 67]. Pregabalin, gabapentin and melatonin [68] can be used as prophylaxis treatment for alleviating postoperative pain [69–71]. Thromboembolic stockings or low-dose subcutaneous unfractionated heparin or low molecular weight heparin (LMWH) can also be  used to prevent the postsurgical development of thromboembolisms [36, 72, 73]. The oral administration of benzodiazepines should also be considered for relieving surgical related anxiety [74].

#### Assessment for required postoperative care

Other factors in addition to obesity may cumulatively determine the extent and the nature of the treatment plan that may be required postoperatively in an obese patient. These factors may include the following: (1) the presence of comorbidities that were present prior to the surgery, (2) an OS-MRS score of 4-5 which indicates an increased risk, (3) the type of surgical procedure applied during surgery, (4) the presence of OSA that is untreated along with an existing need for postoperative opioids administered parentally, and (5) the competence level of the postoperative management team [36]. The type of the surgery and the site at which the surgery was done are both major determinants that influence the degree of postsurgical care that may be required. Patients requiring the administration of long-acting opioids would have to be closely monitored for any complications that may potentially arise [53].

### Intra-operative Care

#### Positioning

In obese patients, excess fat in the cervical region of the neck creates a fat pad causing excessive flexion. Therefore, it is important to elevate the patient’s upper body, head and neck above chest level until the patient’s external auditory meatus lies in the same horizontal plane as the sternal notch [75, 76]. This positioning is called the ramp-up position and it helps to significantly improve intubation outcomes in these patients [76, 77]. The utilization of this position permits better laryngoscopic visualizations in addition to promoting easier ventilation. The ramp-up position may be achieved through the use of folded blankets, pre-manufactured elevation pillows or inflatable pillows [61, 78]. Furthermore, operating tables may be equipped with different features that may facilitate the appropriate positioning of the obese patient with the trunk in an elevated position [75].

#### Intraoperative Fluid Management

During open surgery, due to evaporation, patients potentially experience fluid loss. Obese patients undergoing surgery presents an increased risk for experiencing postoperative renal failure as preoperatively, they commonly present with protracted volume. This protracted volume may be due to prolonged fasting before surgery or due to increased urine output secondary to the use of anti-hypertensive and hypoglycemic drugs. A pre-existing history of renal disease, a BMI greater than 50 kg/m^2^ or extended surgical procedures are all predisposing risk factors [79]. In obese patients, appropriate fluid management is therefore important to prevent renal injury.

One proposed method for fluid management during surgery of morbidly obese patients is to employ a goal-directed therapy (GDT) approach which is guided by the patient’s reaction/ responsiveness to administered fluids [80, 81]. Fluid responsiveness refers to the ability of the heart to respond to an increase in volume through the increase of stroke volume. While maintaining sinus rhythm, fluid responsiveness can be assessed through the analysis of arterial waveforms; a method that provides information on pulse pressure variation (PPV) and stroke volume variation (SVV) [82, 83].

Plethysmographic waveform variation (PWV) provided by the pulse oximetry waveform is also suggested as a useful non-invasive method for determining fluid responsiveness. However, this method has been demonstrated to be more useful at levels of more extreme hypovolemia [84]. The ccNexfin is another non-invasive method that can be used to determine fluid responsiveness through the analysis of CO, PPV and SVV. In obese patients presenting with serious cardiovascular comorbidities, a minimally invasive method called the FloTrac can also be applied for the assessment of fluid responsiveness. With the use of this method, vascular tone and CO can be calculated from the analysis of arterial line waveforms. In addition, it provides information on SVV and when attached to a central venous line, it also gives information on CO and central venous oxygen saturation (ScvO2). During surgery, morbidly obese patients that are deemed high risk can also be monitored using pulse-contour analysis-based techniques, such as PiCCO. In addition to providing information on PPV, SVV and CO, this technology also analyses: (1) Global End-Diastolic Index, (2) intrathoracic blood volume, and (3) extravascular lung water. Despite its usefulness, due to its expensive nature, it is mainly utilized in critically ill patients requiring major surgery [85].

#### Awake tracheal intubation

The use of awake tracheal intubation is one option that can be applied in instances where tracheal intubation seems difficult [86]. Since the presence of obesity is already associated with potential difficult intubations, this method can also be utilized in these patients. With the use of awake tracheal intubation, the upper airway should be appropriately anaesthetized using nerve blocks or aerosolized anaesthetics. Flexible Fiberoptic Bronchoscopy (FOB) and video laryngoscopes are two methods that are incorporated when performing awake intubations. With the patient in the ramp-up position, FOB can be useful for nasal or oral intubations. Excess pharyngeal adipose tissue may make proper visualization difficult with the use of FOB and the placement of the bronchoscope in these situations may further compromise spontaneous breathing. With the use of FOB, laryngeal mask airway may be utilized as means of keeping the airway patent and facilitating breathing after the patient is induced. However, in emergency situations, the use of video laryngoscopes is recommended over FOB [87]. The use of curved blade video laryngoscopes can be applied successfully in obese patients with neck trauma, or in obese patients who are unable to adequately extend their necks or have narrowed oral openings [88]. The use of video laryngoscopes may prove difficult in obese patients presenting with excessive breast tissue [87].

#### Induction and maintenance

Anaesthetic drugs used for inducing non-obese patients can also be used for induction in obese patients. Despite this fact, it is also important to be aware that the presence of excess fat in obese patients affects the pharmacokinetics of anaesthetic drugs depending on their liposolubility and tissue distribution. Obese patients metabolize lipophilic agents more rapidly in comparison to non-obese patients [89].

### Hypnotics

#### Thiopental sodium

Thiopental sodium is a drug that is commonly used for the administration of general anaesthetics. It is highly lipophilic; therefore, an increased volume of distribution is usually observed when it is used in obese patients. Following its administration, the levels of thiopental sodium rapidly decrease in the blood. Thiopental undergoes hepatic elimination and its clearance rate is twice as fast in obese patients as compared to non-obese patients [90, 91].

#### Propofol

Propofol is highly lipophilic; therefore, this anaesthetic agent has a high volume of distribution and is rapidly cleared from the blood following its administration. Due to these features, propofol is the most preferred drug for induction in morbidly obese patients [37, 92]. In obese patients, the administration of continuous infusions of this anaesthetic agent, demonstrates increased volume of distribution and clearance in proportion to total body weight (TBW). One study by Servin et al. that investigated the recovery rates and the pharmacokinetics of propofol infusion in morbidly obese patients, demonstrated that there were no major differences in the initial volume of distribution of propofol in morbidly obese study subjects as compared to non-obese subjects. However, this study also showed that there was a linear increase in volume of distribution at steady state and of clearance with increase in TBW [93].

#### Etomidate

The use of etomidate is recommended in individuals experiencing a state of hemodynamic instability because this drug does not majorly supress the cardiovascular system. However, its use may be of some concern as it has been associated with adrenal insufficiency potentially resulting in organ failure [93, 94]. When used for induction, required dosage adjustments should be made relative to non-fat body weight similar for the pharmacokinetic and pharmacodynamics features observed for propofol and thiopental sodium [37, 92] .

#### Opioids

Obese patients that are undergoing surgery may experience depression of the respiratory system in addition to obstruction of the airway [95]. The use of opioids in the presence of obesity increases the occurrence of obstructive and central sleep apnoea and obese patients may also experience hypoxia and upper airway obstruction [96–98]. As a result, it is important to note that the therapeutic window is narrowed when opioids are used in obese patients.

#### Fentanyl

Fentanyl is one of the most used opioids for anaesthetic induction and it is about 100 times more potent than morphine. The action of fentanyl in the blood is short; however, after continuous infusion, peripheral compartment saturation is achieved [99–101]. This drug is highly lipophilic and therefore has a high volume of distribution. In obese patients, following a single dose of fentanyl, the plasma levels of this drug is significantly reduced as obese patients experience a larger volume of distribution [102]. Fentanyl is cleared at a faster rate in obese patients. There is a non-linear association between clearance of fentanyl and TBW but there is a linear increase in the clearance of fentanyl with “pharmacokinetic mass”, with a significant correlation to lean body weight [103].

#### Alfentanil

As compared to fentanyl, alfentanil is less lipophilic and therefore has a lower volume of distribution. Alfentanil is also less potent as compared to fentanyl. In obese patients, the presence of larger CO significantly decreases plasma levels of alfentanil during early distribution phases. It is therefore theorized that obese patients should experience larger volumes of distribution, longer half-lives and prolonged elimination times of alfentanil as compared to non-obese patients [100, 104].

#### Sufentanil

Sufentanil is more potent than fentanyl and is described as the most lipophilic opioid. Obesity increases the volume of distribution and the rate of elimination of sufentanil but the clearance of this drug in obese patients is comparable to its clearance in non-obese patients [105].

#### Remifentanil

Remifentanil is a rapid acting anaesthetic agent and it is highly metabolized by tissue and plasma esterases thus resulting in a short duration of action in the blood. This anaesthetic agent is normally administered as a continuous infusion when used as a sedative. A combination of remifentanil with inhalation agents or intravenous hypnotic agents can also be used for administration of general anaesthetics [104]. One study aimed at assessing the effects that body weight has on the pharmacokinetics of remifentanil, concluded that there is no significant difference in the observed pharmacokinetics of remifentanil between obese and non-obese patients. This study also concluded that ideal body weight (IBW) or lean body mass should be used to determine the required dose of remifentanil, as the pharmacokinetic parameters of this agent is more closely related to these measurements as opposed to TBW [106]. Another study by Bidgol et al. which compared the use of tight control infusions of sufentanil and remifentanil in morbidly obese patients undertaking laparoscopic gastroplasty surgery, concluded that the use of tight control infusion of sufentanil was associated with better quality of recovery in morbidly obese patients as compared to the use of tight control infusion of remifentanil [107].

### Inhalation agents

The presence of excess fat tissue in obese patients combined with high lipophilicity result in the increased release of inhalation agents. In addition, evidence shows that obese patients take a longer time to recover from anaesthesia due to the prolonged release of inhaled anaesthetic agents from fat tissues [3, 108]. The degree of liposolubility of the different inhalation agents varies and therefore, different agents may exhibit different effects on recovery rates when used in obese patients [109, 110].

#### Isoflurane and Sevoflurane

From the three agents: sevoflurane, desflurane and isoflurane, isoflurane is considered most lipophilic and as a result, this anaesthetic agent is not most favoured for use in morbidly obese patients [111]. In obese patients, there is reduced blood flow to fat tissues, and the time to achieve equilibrium in the blood is usually longer with the use of isoflurane [112, 113] Sevoflurane is not as lipophilic or as soluble as compared to isoflurane; therefore, in morbidly obese patients, the effects of this agent in the blood are usually shorter and it is eliminated more rapid [114]. Despite the lack of evidence to support the exact effects of sevoflurane in patients suffering from renal impairment, this anaesthetic agent should be used with caution in patients suffering from renal insufficiency. One of the metabolic by-products of sevoflurane called inorganic fluoride is toxic to the kidneys at blood concentrations above 50 mmol litre^−1^. Sevoflurane can be broken down into compound A, which may cause renal toxicity [115]. Even though this effect is already proven from animal studies, more evidence is required to determine the effects of compound A on the kidney of humans [116].

#### Desflurane

BMI does not have a significant effect on desflurane’s absorption in the body [111]. Desflurane is the best choice for anaesthetic induction in morbidly obese patients as it is least lipophilic and least soluble as compared to other inhalant agents. Obese and non-obese patients exhibit faster recovery with the use of desflurane as compared to isoflurane [111, 117]; however, evidence comparing recovery rates of desflurane and sevoflurane yields controversial results [118–120].

### Neuromuscular Blockers

Neuromuscular blockers are described as polar and hydrophobic. Due to these properties, these agents are not highly distributed in fat tissues [121].

#### Succinylcholine

Succinylcholine is a non-depolarizing neuromuscular blocker. It is broken down and deactivated by pseudocholinesterase. The levels of pseudocholinesterase are increased in obese patients; therefore, when used during anaesthetic induction in obese patients, the onset and duration of effects of this drug are very rapid and usually, a higher dose of the drug may be needed to produce the required effects. Due to its extremely fast onset of effects and short duration of its action, succinylcholine is preferred for its use in obese patients as these features facilitate prompt tracheal intubations and also promote the rapid restoration of spontaneous ventilation [122, 123].

#### Vecuronium

Vecuronium is a non-depolarizing aminosteroid neuromuscular relaxant. Obese patients show an increase in the volume levels of vecuronium in extracellular fluid; however, this does not affect the volume of distribution of this drug [124]. Vecuronium is removed from the body by the hepatic and biliary systems and improper clearance may prolong the effects of this drug in the body. In addition, when TBW is used for dose estimations, there may be overestimations of required doses, thus resulting in drug overdose. Therefore, in obese patients, the required dose of vecuronium should be calculated based on IBW instead of TBW [124, 125]. Schwartz et al. carried out a study to assess how obesity affects vecuronium with regards to deposition and action. Fourteen participants were recruited; seven obese and seven control subjects. Both groups of patients received 0.1 mg/kg of Vecuronium. This study suggested that when vecuronium is being administered to obese patients, dosage should be calculated based on IBW as it was evident that when calculated based on TBW recovery was delayed due to drug overdose [126].

#### Rocuronium

Rocuronium is described as an aminosteroid neuromuscular blocker which contains a quaternary ammonium group in its chemical structure. Rocuronium is not readily distributed to peripheral tissues and its pharmacokinetics is not majorly affected by the high volumes of extracellular fluid observed in obese patients [127]. In order to prevent the prolongation of the effects of this drug in the body, it is important that the administered dose be calculated based on IBW [122, 128]. Puhringer et al. studied the pharmacokinetics and pharmacodynamics in six obese and six normal weight (control) patients in whom 0.6 mg kg^−1^of rocuronium was administered. It was demonstrated that the time to onset of action of rocuronium was shorter in the obese patients as compared to the control group; however, the duration of action and the time to recovery was comparable for both groups.

### Reversal of Neuromuscular Blocking Agents

Reversal of neuromuscular blockade is a very important phenomenon, especially in obese patients. The presence of obesity is usually associated with an increased risk for the occurrence of respiratory complications following surgery [129, 130]. Obese patients commonly experience a decrease in diaphragmatic tone in addition to a reduction in end-expiratory lung volumes during sleep induction as compared to non-obese patients [131]. The pharmacological reversal of neuromuscular blockage may help to reduce the occurrence of major complications [132].

#### Neostigmine

Neostigmine is described as an acetylcholine receptor blocker. The reversal of neuromuscular blockage with the use of neostigmine has been found to be delayed in patients that are obese. This drug can be administered at dosages between 0.04 and 0.08 mg/kg; however, administered doses should never exceed 5 mg [133].

#### Sugammadex

Sugammadex is a very potent agent used for the reversal of neuromuscular blockage. It is derived from cyclodextrin, has varying degrees of affinity for the different neuromuscular blockers and provides quick and complete recovery from neuromuscular blockage. For the sufficient and total reversal of intermediate or deep blocks, it is recommended that the administered dose of sugammadex be calculated based on TBW or IBW plus 40% [81, 132].

### Postoperative care

Post surgically, obese patients as opposed to non-obese patients possess a higher risk for experiencing respiratory complications such as acute respiratory failure and pneumonia. Lung collapse occurs more often in obese patients following extubation [134, 135]. Patients that are non-obese may experience atelectasis post surgically; however, this condition will rapidly resolve following surgery. On the other hand, in the obese patients, atelectasis takes a longer time to resolve and may result in breathing difficulties post surgically [134]. Once there is awareness, steps can be taken by the postoperative care team to alleviate these potential complications. Postoperatively, obese patients should be closely monitored in the post anaesthesia care unit (PACU) and the following steps should be considered: the patient should be nursed with the head in an upright position [68, 72] and the use of standard oxygen therapy as well as the use of CPAP or non-invasive positive pressure ventilation (NIPPV) should be considered following extubations [36, 74, 136]. High flow oxygen delivered via a nasal cannula may be used [137] and CPAP should also be considered in patients who require opioids [138]. These considerations are important as they help to (1) prevent the occurrence of airway obstruction, (2) ensure proper ventilation, (3) prevent collapse of the lungs, (4) support better gaseous exchange within the lung, (5) restore and preserve normal respiratory functions, (6) improve the patients breathing and (7) reduce the risk for developing postsurgical respiratory failure [139]. Following surgery, the patient should be given oxygen therapy until preoperative arterial oxygen saturation levels are achieved and the patient is totally mobilized. There is an increased likelihood that following surgery, the obese patient will require mechanical ventilation. It is suggested that for mechanical ventilation, peak inspiratory pressure be maintained below 35 cm/H2O and 5–7 ml/kg of tidal volume calculated based on ideal body weight, be administered [140].

Post surgically, it is not recommended that continuous infusions be used for pain management in obese patients requiring opioids. Instead, depending on the procedure performed, opioid analgesics such as fentanyl or morphine can be used for pain control [135]. It is also important to note that myopathies such as rhabdomyolysis can occur in the obese following surgery; therefore, close monitoring is important for the development of deep tissue pains. If post surgically, signs of rhabdomyolysis occur, then steps should be taken to immediately treat this condition and prevent the occurrence of acute kidney injury (AKI) [141]. In addition, evidence suggests that postoperative cognitive dysfunction (POCD) may be a complication observed more commonly in obese patients. Despite the fact that only a minimal association has been established between obesity and this postsurgical complication, it is important to be cognisant of this potential development [142]. Before discharge for care on the surgical ward, it is important that obese patients be monitored for a minimum time of 1 h to ensure that normal respiratory parameters are returned and maintained [135, 143].

## Conclusions

In conclusion, the presence of obesity increases the risk for surgical and postsurgical complications; however, with proper collaborative efforts among medical disciplines, the occurrence of these complications can be reduced quite significantly. It is important preoperatively that certain assessments of the cardiovascular and respiratory systems be carried out. During surgery, proper positioning of the obese patient is very important in addition to the appropriate airway maintenance and fluid management. The choice of anesthetic agent, along with the route of administration is extremely important, as based on their properties, these agents can confer different complications both intra and postoperatively. More intense research is needed for the use of these anesthetic agents in emergency settings. Postoperatively, the necessary steps should be taken to ensure that the patient is fully recovered with limited complications.

## Abbreviations

AKI: Acute kidney injury
BMI: Body Mass Index
CDC: The Centre for Disease Control and Prevention
CO: Cardiac Output
CPAP: Continuous Positive Airway Pressure
DVT: Deep Venous Thrombosis
ERV: Expiratory Reserve Volume
FOB: Flexible Fiberoptic Bronchoscopy
FRC: Functional Residual Capacity
GDT: Goal-Directed Therapy
LMWH: Low Molecular Weight Heparin
MEP: Maximal Expiratory Pressure
MIP: Maximal Inspiratory Pressure
OSA: Obstructive Sleep Apnea
OS-MRS: The Obesity Surgery Mortality Risk Stratification score
PCO_2_: Partial Pressure of Carbon Dioxide
PWV: Plethysmographic Waveform Variation
PPV: Pulse Pressure Variation
POCD: Postoperative Cognitive Dysfunction
PACU: Post Anaesthesia Care Unit
ScvO2: Central Venous Oxygen Saturation
SVV: Stroke Volume Variation
TNF-α: Tumour Necrosis Factor-Alpha
TLC: Total Lung Capacity
TBW: Total body weight
IBW: Ideal body weight
